# Society for the Prevention of Tuberculosis

**Published:** 1901-05

**Authors:** 


### SOCIETY FOR THE PREVENTION OF TUBERCULOSIS.

ON APRIL 18, 1901, the Erie County Society for the Prevention of Tuberculosis was launched under the auspices of members of the medical profession and others, interested in checking the ravages of that most destructive disease.

The organisation was perfected on April 24th, at a meeting held in Mayor Diehl's office. In accordance with the provisions in the constitution and by-laws, adopted on April 18th, the following-named persons were elected as a board of directors to serve for the periods named: Until May, 1902, Mayor Conrad Diehl, Health Commissioner Ernest Wende, Dr. Lee H. Smith, president of the Society of Natural Sciences, Dr. Mary I. Denton, president of the Investigating Club; until May, 1903, Dr. John H. Pryor, Dr. J. C. Thompson,

The Rev. Thomas A. Donahue, D. D., Dr. Albert H. Briggs and Dr. William G. Bissell; until May, 1904, Dr. Benjamin G. Long, the Rev. O. P. Gifford, D.D., Dr. James S. Smith, Dr. Henry R. Hopkins and Dr. Charles E. Congdon.

The lines of work are laid down as follows: (1) by promulgating the doctrine of the contagiousness of the disease; (2) instructing the public in practical methods of its avoidance and prevention; (3) advocating the establishment of institutions for early treatment; (4) co-operating with boards of health in such measures as may tend to the prevention of the disease; (5) advocating the enactment of proper legislation; (6) taking such other measures as may from time to time be deemed necessary to check the ravages of tuberculosis.

The Erie County society is local in scope and is the first of its kind to be formed in this state; an organisation of a similar nature in Philadelphia has done much to prevent the spread of consumption. The membership is not to be confined to the medical profession, as any person who pays $1.00 or more, is a member for the year in which the payment is made. It is the desire, however, of the promotors, that the medical profession shall ally themselves with this work to a man.

Meetings of the members are to be held semi-annually, while the directors are to meet monthly. Any one who wishes to assist in this meritorious work, can send his name and $1.00 or more to the treasurer, Dr. A. H. Briggs, 267 Hudson Street, and a receipt will be mailed to him.

Bulletins and other matter issued by the society will be placed in the hands of the members and they will be urged so far as possible to assist in the education of the public along the lines of prevention. The movement starts off well with an enthusiastic board of directors, whose aim is to do much good for our fair city.

The following well-known physicians were elected officers for the ensuing year.

President, Dr. Benjamin G. Long; vice-president, Dr. Henry R. Hopkins; secretary, Dr. William G. Bissell; treasurer, Dr. Albert H. Briggs.

